# COVID-19 and Mass Fatality Management: A Public Health Challenge

**DOI:** 10.1017/dmp.2020.277

**Published:** 2020-07-27

**Authors:** Anant Kumar, K. Rajasekharan Nayar

**Affiliations:** Xavier Institute of Social Service, Ranchi, India; Global Institute of Public Health, Thiruvananthapuram, India

**Keywords:** COVID-19, dead body, management, mass fatality, pandemic, public health

## Abstract

The COVID-19 pandemic has posed a serious question over preparedness to deal with mass fatality. The current trend shows that there would be more bodies than the capacity and resources to handle them. The international agencies have alerted governments that the number of deaths may overwhelm the local capacity to handle dead bodies properly. Mass fatality management and planning are important to respecting the dignity of the deceased and surviving family. Inadequate capacity to deal with dead bodies may affect the psychological well-being of survivors which may result in distress to families and community.

The care with which our dead are treated is a mark of how civilized a society we are.

– Charles Haddon–Cave

The coronavirus disease (COVID-19) pandemic has become a global threat with uncertainty related to virus behavior, its nature, and spread. Scientists are struggling to understand the virus’s behavior and to develop treatment protocols. In the absence of medicines and vaccines, the fatality rate is rising and, so far, over 600 000 people worldwide have lost their lives.^1^

Unofficial estimates indicate that the death toll might rise multifold globally. In such a scenario, local bodies, state, and governments along with the private sector and faith-based community organizations need to evolve mass fatality management plans for handling the dead bodies, as well as for their disposal.^2^ It is important to help and lower the pain that families and broader society feel in the face of a high death toll. It may be distressing and heartrending when mass fatalities are mismanaged accompanied by grave emotional and mental health consequences that can delay recovery and undermine community resilience.

The pandemic poses a question over preparedness to deal with mass fatalities, particularly the management and cremation/burial of dead bodies. Although there is no unanimity over whether COVID-19 human remains are infectious, the likelihood is high. The World Health Organization (WHO) warns that corpses can spread symptoms to living people. In some cases, due to heightened fear, dead bodies were mistreated in the morgue, dragged by the morgue employees by the neck with tongs at a crematorium.^3^ In Italy and other countries, many communities did not get an appropriate place to bury their loved ones due to a lack of space in cemeteries. The lack of space in the graveyards is forcing Muslims and Christians to pay premium prices or to move to the outskirts of the city to bury their loved ones.^4^

The management of the dead bodies in a proper and dignified manner is one of the most difficult aspects of pandemic response, which is also related to the management of dead bodies, their identification, storage, and disposal. Despite several pandemics and deaths in the past, many countries across the globe do not have a policy, fatality infrastructure, and preparedness plans for the management and cremation of bodies. The absence of a policy and a plan has led to considerable delays where dead bodies were waiting for last rites due to a lack of preparedness.

Governments should learn from this pandemic and prepare a mass fatality plan and policy for handling the bodies with a focus on operational procedures for their recovery, storage, and identification; a medico-legal investigation protocol; funeral services; and final disposition. Considering the global, multi-ethnic, and religious dimensions of the pandemic, sensitivities should be maintained and addressed appropriately. The WHO has suggested guidelines to manage dead bodies, but given the ethnic and religious complexities, they partially address the problem.^5^ Governments should realize and understand that the inadequate capacity to deal with dead bodies may affect the psychological, spiritual, and mental well-being of survivors.
